# Use, acceptability and impact of booklets designed to support mental health self-management and help seeking in schools: results of a large randomised controlled trial in England

**DOI:** 10.1007/s00787-016-0889-3

**Published:** 2016-07-21

**Authors:** Helen Sharpe, Praveetha Patalay, Panos Vostanis, Jay Belsky, Neil Humphrey, Miranda Wolpert

**Affiliations:** 1 Department of Clinical Psychology, University of Edinburgh, Teviot Place, Edinburgh, EH8 9AG UK; 2 Evidence Based Practice Unit, University College London and the Anna Freud Centre, London, UK; 3 Centre for Longitudinal Studies, UCL Institute of Education, London, UK; 4 Department of Neuroscience, Psychology and Behaviour, University of Leicester, Leicester, UK; 5 Department of Human Ecology, University of California, Davis, USA; 6 Manchester Institute of Education, University of Manchester, Manchester, UK

**Keywords:** Self-management, Help seeking, Schools, Adolescents, Mental health promotion

## Abstract

Mental health booklets may provide a low-cost means of promoting mental health self-management and help seeking in schools. The aim of the study was to assess the (a) use, (b) acceptability and (c) impact of booklets for students in primary (10–11 years) and secondary school (12–13 years) alone and in conjunction with funding for targeted mental health support. This was a 2 × 2 factorial cluster randomized controlled trial, in which 846 schools in England were randomly allocated to receive/not receive: (1) booklets for students containing information on mental health self-management and help seeking, and (2) funding for mental health support as part of a national mental health initiative. 14,690 students (8139 primary, 6551 secondary) provided self-report on mental health, quality of life (baseline and 1 year follow-up) and help seeking (follow-up). (a) Approximately, 40 % primary school students and 20 % secondary school students reported seeing the booklets. (b) Of these, 87 % of primary school students reported that the booklet was ‘very helpful’ or ‘quite helpful’, compared with 73 % in secondary school. (c) There was no detectable impact of booklets on mental health, quality of life or help seeking, either alone or in conjunction with additional funding through the national mental health initiative. Lack of discernable impact of booklets underscores the need for caution in adopting such an approach. However, it is feasible that the impact was obscured by low uptake or that booklets may be more effective when used in a targeted way.

**Electronic supplementary material:**

The online version of this article (doi:10.1007/s00787-016-0889-3) contains supplementary material, which is available to authorized users.

## Introduction

Mental health problems in childhood and adolescence are associated with low quality of life [1] and poor academic achievement [2], and forecast longer-term difficulties, including mental health problems throughout adulthood [3, 4]. Half of all lifetime mental illness has an onset by age 14 [5], making the pre- and early adolescent years particularly pivotal for intervention.

Helping to support positive mental health and resilience in schools is a key priority [6], and finding low-cost means to do so is a particular imperative in times of austerity and cuts in services. Mental health promotion activities in schools––particularly those targeted at children at risk of mental health problems––have been shown to be effective in improving well-being and mental health, although the quality of evaluations in schools is recognised as being poor [7]. Access to these types of interventions may be hindered by low mental health literacy and stigmatising attitudes towards mental health problems [8]. The provision of booklets and other forms of information have been recommended as potential low-cost routes for promoting improved recognition of mental health symptoms in young people [9]. Increased knowledge about mental health problems has also been shown to be associated with improved self-management, such as appropriate help seeking [8, 10, 11]. Given the potential for wide dissemination of information booklets, even very small effects of this type of intervention may translate into meaningful impact on mental health and well-being at the population level. However, rigorous evaluation is needed to determine whether this is indeed the case.

As part of a major government-sponsored initiative across England to support improved mental health provision in schools, the UK government implemented the £60 million Targeted Mental Health in Schools (TaMHS) programme in 2008. Selected schools in every local (government) authority (LA) in England were funded to provide targeted support for children at risk of developing mental health problems. The initiative did not stipulate how the funds were to be allocated, as long as local programmes were in line with two core principles: choosing interventions informed by evidence, and promoting strategic integration across agencies. As such, a wide range of interventions were implemented, including peer support, individual therapy, training and information for parents, and training and support for school staff [12, 13]. The impact of the TaMHS programme was evaluated using a randomized controlled trial (RCT), in which LAs were randomly allocated to immediate TaMHS provision or to a one-year wait-list control condition. Results of the RCT indicated that the TaMHS group had better behavioural outcomes in primary school than the wait-list controls, though no such group differences emerged for emotional outcomes or on any outcomes in secondary schools [13].

Given the known barriers to children accessing mental health interventions [8], this study also examined whether there might be ways to augment the impact of this targeted support. In addition to the main RCT, schools were randomly allocated to receive student booklets that were designed to increase basic psychoeducation and mental health awareness, to draw on evidence about simple self-management approaches, as well as to provide information about how to access support. The self-management content drew on principles and activities from low-intensity evidence-based interventions, including cognitive-behavioural therapy [e.g. 14] and positive psychology interventions [e.g. 15]. For example, activities delineated in the booklets included ways to relax when feeling stressed, such as progressive muscle relaxation, and listing, in writing, three good things that happen each day. These activities were designed to be applicable universally, meaning that the booklets could be used as a general mental health promotion tool, or employed in a more targeted way with children facing particular challenges.

**Fig. 1:**
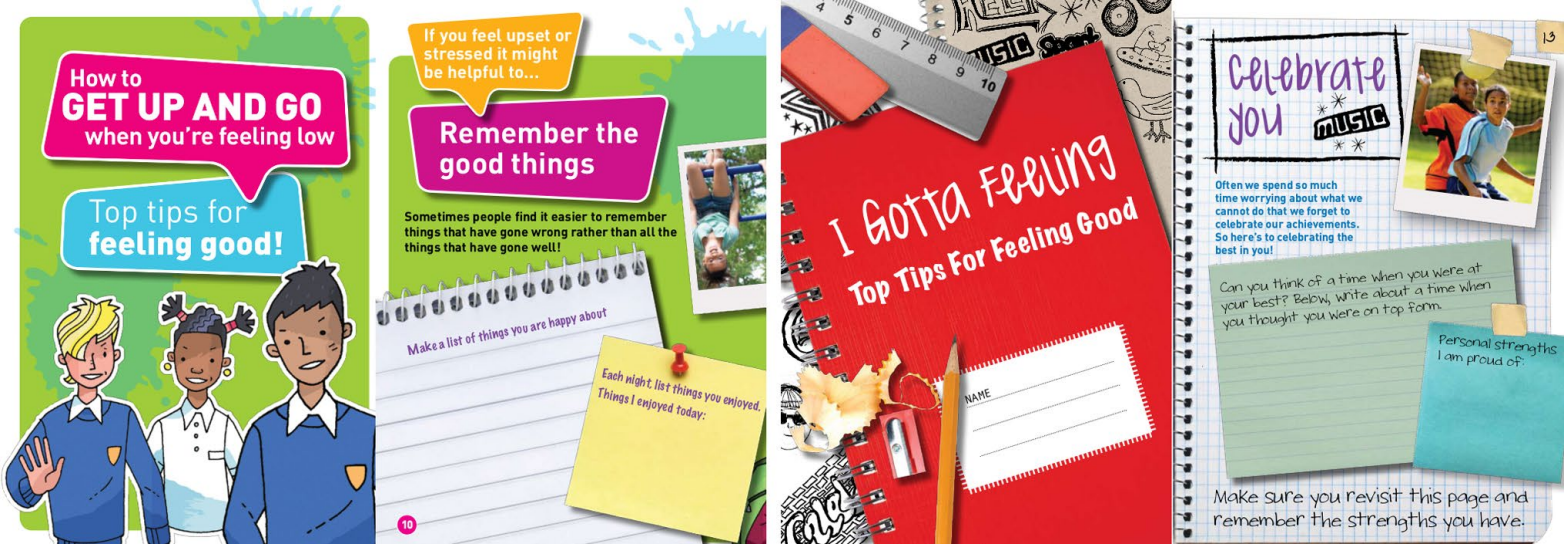
Example pages from the intervention booklets, “*How to Get Up and Go When You’re Feeling Low”* (*left panels*) and “*I Gotta Feeling”* (*right panels*). Copies of the booklets are available from the authors

Two booklets (see Fig. 1) were created with young people’s input on content and design: “*How to Get Up and Go When You’re Feeling Low”* was aimed at young people in primary school (aged 8–11) and “*I Gotta Feeling”* was aimed at young people in secondary schools (aged 11–14). The hypothesis was that these booklets would themselves increase help seeking (i.e. direct effect) and/or amplify positive effects of TaMHS provision on help seeking in those schools in which the availability of specialist support was being improved (i.e. interactive effect). Thus, the 2 × 2 factorial nature of this RCT design (i.e. TaMHS/no TaMHS and booklets/no booklets) positioned us to evaluate the impact of these booklets, both independently and in conjunction with TaMHS provision [16]. The aim of the research reported herein was, therefore, to answer the following research questions:

1. *Use*: Did school students report having seen the booklets?
2. *Acceptability*: Did school students view the booklets as being helpful?
3. *Impact*: Did the booklets (a) promote mental health, quality of life or help-seeking behaviour and/or (b) enhance the efficacy of TaMHS provision?

## Methods

### Trial design

This study was a hierarchical cluster randomised control trial with a 2 × 2 factorial design. Participants were clustered within schools and then within LAs. Randomisation occurred in two stages: first, LAs were randomised in a 1.5:1 allocation to receive or not to receive TaMHS funding; second, schools within those LAs were randomized in a 1:1 allocation to receive or not to receive booklets. The trial protocol was not registered, but details of the full protocol, including outcomes not addressed in this manuscript, are reported elsewhere [12, 13].

### Procedure

Ethical permission for the study was granted by the University College London Research Ethics Committee. Randomisation by random number generator was conducted independently from the research team that enrolled participants and carried out the analysis. Assessments were completed by students at baseline (prior to school level randomization) and post-intervention (1 year later). At all assessment points, students were blind to their condition. Parental consent (opt out) and student assent (opt in) were sought prior to each data collection point. Students completed assessments using a secure online system during their usual school day. Teachers facilitated the completion of the survey by reading a standardised information sheet to participating children outlining what the questionnaire was about, the confidentiality of their answers and their right to decline participation.

### Participants

Participants were students from primary to secondary schools in England. Students were eligible to participate if they attended year 4 (age 8–9) or year 7 (age 11–12) in a participating school, had parental consent and provided assent. Participating schools were selected by the 75 LAs taking part in the TaMHS initiative (i.e. not by the evaluation team). The only inclusion criterion was that schools be state funded.

**Fig. 2:**
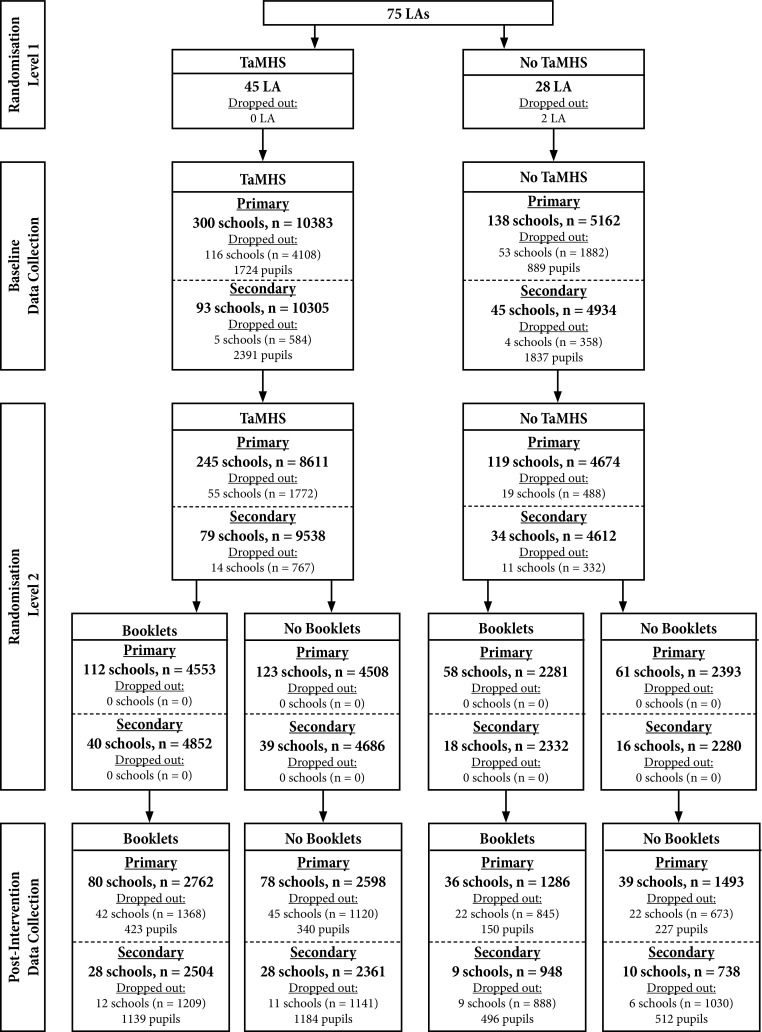
CONSORT diagram showing flow of participants through the trial

Figure 2 outlines the flow of participants through this trial. Of the 75 LAs involved, 45 were allocated to receive TaMHS funding, with the remaining 30 forming a one-year wait-list control group. Two LAs from the wait-list control arm dropped out of the trial at this point. After baseline assessment in 2009, participating schools could then opt out from school-level randomisation to further conditions. Hence, 486 schools were randomly allocated to one of the two booklets conditions. This resulted in four arms of the current evaluation, with schools receiving (1) both TaMHS and booklets (TaMHS + booklets, 162 schools), (2) just TaMHS (TaMHS only, 162 schools), (3) just booklets (Booklets only, 76 schools) and (4) neither TaMHS nor booklets (No intervention, 77 schools). As can be seen in Fig. 2, 8139 primary school students and 6551 secondary school students provided both baseline and post-intervention assessments and were, therefore, included in the analysis. No data were collected regarding reason for drop out/non-response from schools or students.

Of these 8139 primary school participants, 49.8 % were female, 74.3 % White, 10.9 % Asian, 7.7 % Black, 4.6 % Mixed and 2.5 % other ethnic groups or unclassified. Compared with 2009 national averages for primary school students [17], this sample slightly over-represented Black students (national average = 4.9 %) and under-represented White students (national average = 79.2 %). As a measure of economic deprivation, 24.8 % were eligible for free school meals (FSM), as compared with the 16.0 % national average for this population [17].

Of the 6551 secondary school students included in analysis, 48.9 % were female, 80.7 % White, 6.0 % Asian, 6.6 % Black, 4.5 % mixed, 2.3 % other ethnic groups or unclassified and 18.8 % FSM eligible. The proportion of participants in different ethnic groups is within 2 % of 2009 national norms for secondary school students [17]. The sample, however, over-represented children eligible for FSM (national average = 13.3 %) [17].

### Interventions

#### TaMHS provision

As described elsewhere [13], TaMHS provision consisted of funding and support to enhance the existing provision for mental health support in schools. The funding could be used in different ways (e.g. to fund training, recruitment of staff), as determined by local agreement, and in accordance with principles of evidence-based practice (though this was not monitored) and organisational collaboration [13].

#### Booklets

Booklets (see Fig. 1) were sent to an identified pastoral lead in those schools assigned to the booklet condition, along with general advice on how they could be used and the age group for which they were relevant. An electronic version of the booklets was also provided to schools in order to facilitate use in classrooms. Confirmation was received from schools on receiving the booklets. The schools were issued guidance to distribute and use the booklets as they deemed best, including placement in the school library, distribution in class or incorporation into relevant lessons such as personal, social and health education. Although students were asked whether they saw the booklets (to assess uptake, see below), it is important to note that we do not know whether or how they were distributed in school, or the context in which they were used. Copies of the booklets are available at http://www.ucl.ac.uk/ebpu/publications/children and hard copies can be requested from http://ebpu@annafreud.org.

### Measures

#### Demographic information

Student ethnicity, gender and free school meal eligibility, the latter serving as a measure of deprivation, were obtained from the National Pupil Database and matched to self-report outcomes. Once matched, all data were anonymised.

#### Booklet use

Uptake of the booklets was assessed at post-intervention. Students were shown pictures of five booklets and asked to report if they had seen them. The five booklets included: (1) the primary and secondary intervention booklets from this trial, (2) two other genuine booklets available in the UK but not distributed as part of the current evaluation study (*Young Minds* booklet and *Take Action* booklet), and (3) a sham booklet (that did not exist) constructed for the purposes of determining uptake.

#### Booklet acceptability

If a student endorsed having seen a booklet, s/he was asked whether s/he had found the booklet to be very helpful, quite helpful or not helpful.

#### Booklet impact

Mental health, quality of life and help seeking were assessed to evaluate impact. These were measured pre- and post-intervention.

#### Mental health

Mental health was assessed at pre- and post-intervention by completion of the Me and My School Questionnaire [18, 19], which consists of a 10-item emotional difficulties subscale (e.g. “I worry a lot”, alpha = 0.70−0.77) and a 6-item behavioural difficulties subscale (e.g. “I get very angry”, alpha = 0.78−0.80). Students responded to each item by endorsing the response options: never, sometimes, always. Validation studies of the Me and My School Questionnaire, which was developed for the TaMHS evaluation, provide evidence of its robust psychometric properties [18, 19].

#### Quality of life

Quality of life was assessed at pre- and post-intervention, using nine of 10 items from the self-report KIDSCREEN-10 measure [20]. KIDSCREEN-10 measures health-related quality of life and includes items such as “have you felt fit and well” and “have you been able to do the things that you wanted to do in your free time”. (One excluded item concerned parental relations and home life: “Have your parent(s) treated you fairly?”). The full KIDSCREEN-10 has strong psychometric properties in large studies of European children and adolescents [20]. In these samples, internal consistency of the nine item measure was 0.73–0.77. In the current sample, it ranged from 0.75 to 0.78 across measurement occasions.

#### Help seeking

Helpseeking behaviour was assessed using three items at post-intervention, asking students how frequently they sought help in the past year, because they had been ‘sad, stressed or angry’, from (1) a counsellor, (2) a peer mentor or (3) another source in school. Responses were on a four-point scale (never, once, a few times, more than five times).

### Statistical methods

All analyses were conducted using STATA12 [21]. Use and acceptability of booklets were examined using chi squared analysis comparing the likelihood of (a) seeing the booklets and (b) finding them helpful across the four conditions. Impact outcomes were examined using mixed-effects models that accounted for the nested structure of the data (i.e. students in schools, schools in LAs). In addition to the main effects of the intervention condition, the models included the baseline score for the efficacy outcome and socio-demographic variables that varied between the arms (see Table 1). Impact on help seeking was examined using logistic mixed-effects models (never sought help vs. sought help at least once). All analyses were based on intention-to-treat (i.e. based on assigned arms rather than receipt of the intervention), and alpha was set to 0.01 in light of the large sample size.

**Table 1:** Baseline characteristics of the samples in the different arms of the study

	Primary school					Secondary school				
	TaMHS + booklet	Booklet only	TaMHS only	No intervention	Test (*df* = 3)	TaMHS + booklet	Booklet only	TaMHS only	No intervention	Test (*df* = 3)
Gender (% female)	50.2	49.1	49.8	50.2	*χ* ^2^ = 0.76, *p* = 0.86	50.8	46.9	42.5	56.8	*χ* ^2^ = 41.24, *p* < 0.001
Ethnicity (% White)	72.9	75.3	71.1	77.7	*χ* ^2^ = 20.35, *p* < 0.001	83.0	81.0	83.5	68.4	*χ* ^2^ = 84.83, *p* < 0.001
Deprivation (% FSM eligible)	22.1	29.1	26.5	20.7	*χ* ^2^ = 52.52, *p* < 0.001	19.8	18.1	13.4	24.5	*χ* ^2^ = 36.34, *p* < 0.001
Emotional problems [m (sd)]	6.90 (3.47)	6.87 (3.52)	6.74 (3.38)	6.84 (3.52)	*F* = 0.63, *p* = 0.60	5.67 (3.34)	5.38 (3.22)	5.79 (3.43)	5.65 (3.48)	*F* = 4.77, *p* = 0.003
Behavioural problems [m (sd)]	3.10 (2.50)	3.20 (2.62)	3.18 (2.56)	3.09 (2.50)	*F* = 1.02, *p* = 0.38	3.26 (2.43)	3.16 (2.40)	3.29 (2.40)	3.32 (2.52)	*F* = 1.37, *p* = 0.25
Quality of life [m (sd)]	31.37 (4.75)	31.42 (4.86)	31.58 (4.71)	31.30 (4.71)	*F* = 0.87, *p* = 0.46	30.68 (4.20)	30.62 (4.24)	30.21 (4.23)	30.75 (4.38)	*F* = 3.16, *p* = 0.023

As outlined in the introduction, because the TaMHS provision was specifically targeted at those experiencing or at risk of mental health problems, we also conducted sensitivity analyses to explore whether effects were different for children above the at-risk threshold for mental health symptoms (as defined by being above the cut-off on either scale of the pre-intervention mental health measure).

To examine missing data, we compared baseline characteristics of those pupils who did and did not provide data at post-intervention. Analyses indicated that drop out disproportionately affected pupils from more deprived backgrounds in secondary schools [OR = 0.88 (0.81–0.96), *p* = 0.003], and pupils with greater behavioural difficulties in both primary and secondary schools [primary: OR = 0.98 (0.97–0.99), *p* < 0.07; secondary: OR = 0.96 (0.95–0.98), *p* < 0.001]. Mixed-effects models are robust to missing data that are missing at random if variables related to missingness are included in the models [22]. Inclusion of these variables in the models did not alter the results, so for clarity unadjusted models are presented herein.

## Results

### Baseline demographic characteristics

Table 1 outlines the distribution of students in the four arms, based on their socio-demographic characteristics. Comparisons between the arms show that, in spite of randomisation, there were significant differences between arms on key demographic variables. These distinguishing variables were, therefore, controlled in all analyses. In terms of mental health and quality of life outcomes, there were no differences between conditions in the primary sample, whereas in the secondary school sample there were small differences in emotional symptoms at baseline (see Table 1). Given that baseline scores are included in the statistical models, this imbalance is accounted for.

### Booklet use

Significantly more students reported seeing the two intervention booklets in the arms where they were available (TaMHS + booklet, Booklet only) compared with the non-booklet arms (TaMHS only, No intervention). In the primary school sample, ~40 % of the students reported seeing the intervention booklets in the booklet arms (TaMHS + booklet = 39.4 %, Booklet only = 41.5 %); these rates are significantly higher than the 12.8 and 9.3 % in the non-booklet arms (*χ* ^2^(1) = 864.84, *p* < 0.001). Similarly, although overall proportions are lower in secondary schools, the same pattern emerged: 18.4 and 21.6 % of students reported seeing the booklets in the TaMHS + booklet and Booklet-only conditions, respectively, whereas only 4.3 and 5.5 % reported seeing the booklet in the non-booklet arms. Once again these rates were found to be significantly different (*χ* ^2^(1) = 323.31, *p* < 0.001).

In contrast to the intervention booklets, there was no difference between the conditions for participants seeing the sham booklet: in primary schools, the percentage who reported this ranged from 9.9 to 11.9 % across conditions (*χ* ^2^(1) = 1.79, *p* = 0.18) and in secondary schools from 5.1 to 6.3 % across conditions (*χ* ^2^(1) = 0.17, *p* = 0.68). These proportions are similar to those for children who reported seeing the booklets in the non-booklets condition, and suggest that only a small proportion of students responded in affirmation to all the booklets in the survey.

In the primary school sample, participants that were at risk of mental health problems were no more likely than low-risk participants to report seeing the intervention booklet (*χ* ^2^(1) = 1.80, *p* = 0.18). In contrast, in the secondary school sample, participants that were at risk of mental health problems were more likely to report seeing the intervention booklet compared with the low-risk students (*χ* ^2^(1) = 6.86, *p* = 0.009). It worth noting, however, that the absolute differences were small: in the booklets arms (TaMHS + booklet and Booklet-only), 22.3 % of at-risk participants reported seeing the intervention booklets compared with 18.3 % of low-risk participants.

### Booklet acceptability

Responses to perceived helpfulness of the booklets are reported for those participants in the booklet conditions (TaMHS + booklet, Booklet only) who reported seeing the intervention booklets. In primary schools, 41.4 % of these children found the booklets to be very helpful, followed by 46.0 % finding them quite helpful and 12.6 % finding them not helpful. In the secondary school sample, utility was less favourable: 16.6, 56.7 and 26.7 % found the booklets to be very, quite and not helpful, respectively. These proportions did not differ based on condition (i.e. TaMHS + booklet vs. Booklet only).

Children that were at risk of mental health problems were less likely to report that the booklet was helpful compared with low-risk children (focusing again on just those children in the booklet condition who reported seeing the booklet). In primary school, 17.1, 43.0 and 39.9 % of at-risk children reported that the booklet was not helpful, quite helpful and very helpful, respectively, compared with 10.4, 47.5 and 42.1 %, respectively, in the low-risk group. These group differences were significant (*χ* ^2^(2) = 14.68, *p* = 0.001). Similarly, in secondary school, 36.5, 51.0 and 12.5 % of at-risk children reported that the booklet was not helpful, quite helpful and very helpful, respectively, compared with 22.5, 59.2 and 18.4 % in the low-risk group. Once again these group differences proved significant (*χ* ^2^(2) = 14.85, *p* = 0.001).

### Booklet impact

Tables 2 and 3 present the results of models comparing booklets to no booklets on measures of impact. (Descriptive statistics for impact outcomes at post-intervention are provided in supplementary materials in Table S1.) As can be seen from the estimate for ‘condition’ in Table 2, the presence of booklets was *not* associated with any differences in mental health or quality of life in either age group. There was also no difference in help-seeking behaviour (Table 3). Sensitivity analyses focusing specifically on children scoring above the at-risk threshold at baseline produced the same (null) results. (Details of models available from the authors.)

**Table 2:** Impact of booklets on mental health and quality of life

Parameter	Primary school			Secondary school		
	Emotional	Behavioural	Quality of life	Emotional	Behavioural	Quality of life
	Estimate (SE)	Estimate (SE)	Estimate (SE)	Estimate (SE)	Estimate (SE)	Estimate (SE)
Intercept	2.73*** (0.11)	1.79*** (0.07)	22.00*** (0.36)	1.84*** (0.11)	1.48*** (0.09)	18.60*** (0.38)
Baseline score	0.46*** (0.01)	0.46*** (0.01)	0.29*** (0.01)	0.53*** (0.01)	0.55*** (0.01)	0.38*** (0.01)
Gender (female)	0.61*** (0.07)	−0.67*** (0.05)	−0.17 (0.10	0.35*** (0.07)	−0.26*** (0.05)	−0.36*** (0.10)
Free school meals (yes)	0.25** (0.08)	0.34*** (0.06)	−0.37** (0.13)	0.38*** (0.10)	0.35*** (0.07)	−0.27* (0.13)
Ethnicity (Asian)	0.18 (0.13)	−0.24** (0.09)	0.57** (0.19)	−0.17 (0.16)	−0.12 (0.11)	−0.06 (0.22)
Ethnicity (Black)	0.03 (0.14)	0.25** (0.09)	−0.12 (0.21)	−0.29 (0.16)	−0.07 (0.12)	0.12 (0.22)
Ethnicity (mixed)	−0.11 (0.17)	0.04 (0.11)	−0.08 (0.25)	−0.01 (0.18)	0.13 (0.12)	−0.20 (0.24)
Ethnicity (other)	0.18 (0.23)	−0.14 (0.15)	0.34 (0.35)	−0.06 (0.24)	−0.15 (0.17)	−0.01 (0.33)
Condition (booklet)	−0.02 (0.11)	0.03 (0.07)	0.07 (0.14)	0.05 (0.12)	0.11 (0.10)	−0.13 (0.16)

*** *p* < 0.001, ** *p* < 0.01

**Table 3:** Impact of booklets on help seeking

Parameter	Primary school			Secondary school		
	Counsellor	Peer mentor	Other help	Counsellor	Peer mentor	Other help
	Estimate (SE)	Estimate (SE)	Estimate (SE)	Estimate (SE)	Estimate (SE)	Estimate (SE)
Intercept	−0.28*** (0.07)	−0.55*** (0.06)	0.33*** (0.06)	−1.35*** (0.09)	−1.54*** (0.10)	−0.91*** (0.08)
Gender (female)	−0.16*** (0.05)	−0.17*** (0.05)	−0.27*** (0.05)	−0.18** (0.06)	−0.22*** (0.07)	−0.20*** (0.06)
Free school meals (yes)	0.28*** (0.06)	0.25*** (0.06)	0.27*** (0.06)	0.59*** (0.08)	0.44 *** (0.08)	0.44*** (0.07)
Ethnicity (Asian)	−0.03 (0.09)	0.05 (0.09)	0.17* (0.09)	−0.02*** (0.14)	−0.24 (0.16)	−0.15 (0.13)
Ethnicity (Black)	−0.01 (0.10)	−0.11 (0.10)	0.08 (0.10)	0.11 (0.14)	0.01 (0.15)	−0.10 (0.13)
Ethnicity (mixed)	−0.09 (0.12)	−0.11 (0.12)	−0.01 (0.11)	0.12 (0.15)	0.00 (0.16)	−0.01 (0.14)
Ethnicity (other)	−0.05 (0.16)	−0.06 (0.16)	0.10 (0.16)	−0.47* (0.23)	−0.17 (0.23)	−0.08 (0.19)
Condition (booklet)	−0.11 (0.08)	0.09 (0.255)	0.01 (0.06)	0.15 (0.11)	0.22 (0.13)	0.11 (0.10)

*** *p* < 0.001, ** *p* < 0.01

To determine if the presence of booklets enhanced the efficacy of TaMHS provision, we tested whether including a TaMHS × booklet interaction in the model significantly improved the prediction of the efficacy outcomes (i.e. emotional problems, behavioural problems, quality of life and helpseeking behaviours). These analyses revealed no significant interactions for any efficacy outcome. (Full details of these models available from the authors.) Hence, there was no evidence that booklets enhanced––or undermined––the efficacy of the TaMHS provision. Sensitivity analysis focused on pupils above the at-risk threshold at baseline produced identical results (i.e. no interaction between TaMHS provision and booklets for any efficacy outcome).

## Discussion

This study is the first of its kind to involve a rigorous randomised control trial of an approach that is often suggested in the school context: the dissemination of written materials to support self-management and appropriate help seeking in young people experiencing mental health problems. The key finding––that there was no discernable impact of the booklets on mental health, quality of life or help seeking––is of relevance when considering provision of resources to support such an approach and may indicate the need for caution in recommending it. Given the low intensity of this intervention, the lack of impact for booklets alone is perhaps unsurprising. However, there was also no evidence that the booklets enhanced the previously documented mental health benefits of the broader TaHMS initiative [13]. This suggests that, even as an adjunct to more extensive and targeted support, the benefit of information booklets for mental health may be minimal and undetectable. Fortunately, no negative impact was discerned either.

Self-management or ‘informal self-help’ has been proposed as part of an ‘overlapping waves of action’ model, in which the likelihood of accessing different types of support (ranging from informal self-help strategies, such as seeking support from friends, through to engagement with professionals) varies with increasing levels of psychological distress [23]. The booklets evaluated in this study aimed to promote such self-management by drawing on evidence-based approaches to promoting well-being. The lack of impact suggests that using booklets as a medium to convey these self-management strategies is not effective.

We should temper our conclusions by highlighting the fact that we did not have direct records regarding booklet usage. An unfortunate, but necessary, compromise in a study of this scale was the limited depth of data collected from any school or individual child. This means that the current pragmatic study design cannot untangle the impact of uptake or the type of use from the efficacy of the materials themselves. Although students were significantly more likely to report seeing the booklets in the schools where they were provided, we do not know whether the booklets were read or the information in them was used. It may be that the impact of the booklets by those who used them was obscured by limited usage overall. It is also plausible that the booklets would be more effective when used in a targeted way (e.g. with the support of the pastoral care team) rather than simply being made available for students to pick up. Whilst there were no differences in primary schools, in secondary schools, children at risk of mental health problems were slightly more likely to report seeing the booklets compared with low-risk children. However, the absolute differences between the arms were small, thus suggesting that in general the booklets were not being used in a targeted way. Given that we do not have information on how the booklets were used, the results of this trial do not preclude that booklets of this kind *could* be beneficial when used in particular ways (e.g. in some contexts, with some pupils). However, the results do suggest that a universal, non-directed delivery (i.e. sending booklets to schools to be used however is deemed appropriate) is unlikely to be a fruitful approach.

A second notable finding was that there were clear differences in the uptake and acceptability of the booklets between the primary and secondary school samples. Recall that children in primary school were both more likely to report having seen the booklet, implying perhaps that they were being used more widely, and more likely to endorse the booklets as being helpful compared with their secondary school counterparts. This finding may reflect age differences in responses biases (e.g. potentially younger children are biased towards providing favourable feedback), but could suggest that a booklet-based approach is more suitable for this younger-age group. It could be that younger children are more open to the medium generally, that they liked the content of this booklet specifically, or that the booklets were being used in a different way with younger children that favourably impacted acceptability. In both the primary and secondary samples, the booklets were less likely to be viewed as being helpful by the children at risk of mental health problems. There would seem to be a need for better understanding of what types of support materials might be suitable and acceptable for different children, and whether this finding is specific to the booklets evaluated here or applies more generally.

This study builds on existing work in a number of ways. First, the use of a rigorous study design and very large sample size provides the statistical power to detect small effects that perhaps should be expected from a universal intervention approach such as information provision. It is thus unlikely that the reported null findings reflect a lack of power. Second, rather than examining changes in knowledge [e.g. 9], we focused on mental health symptoms, quality of life and helpseeking behaviour. This was a conservative approach, as changes in knowledge may take time to translate into behaviour change, but one which does focus on clinically salient outcomes.

Future work could helpfully explore the potential impact of other low-cost, high-volume initiatives, such as information booklets, on a broader range of outcomes, including increasing awareness and understanding of mental health difficulties, which were not the focus of the current study; utilization of new technologies such as social media is also worth considering. Ensuring that it is possible to monitor the uptake and use of these types of interventions will be essential for interpreting findings (especially null results). It may be worth nesting smaller, more focused, studies within larger evaluations to explore how materials are actually being put into use. In line with calls to better assess harms in evaluations of psychological interventions [24], future work should also assess a wider range of potential harms, such as stigmatisation or bullying. Given the disparity between perceived helpfulness and the efficacy outcomes, at least in the primary school sample, it would also be useful to conduct a more in-depth study examining students’ views on the booklets than was possible in the current research. This could provide clearer indication as to what underlies the lack of impact in this evaluation. Finally, as noted above, it will be important to explore whether we can identify approaches or contexts in which the use of information booklets is useful, such as when being used in collaboration with pastoral staff, or as part of a broader health promotion curriculum.

## Conclusions

Results of this RCT suggest that the widespread provision of information booklets aiming to increase self-management and help seeking for mental health problems in young people is not an effective strategy when booklets are simply made available in schools. Instead, alternative low-cost approaches need to be explored. We hope this work will stimulate future rigorous evaluation of similar interventions, to ensure that what appear to be low-cost, high-volume initiatives are not rolled out without suitable evidence for their effectiveness.

## Electronic supplementary material

Below is the link to the electronic supplementary material.

Supplementary material 1 (DOCX 73 kb)

## References

[1] Sawyer MG, Whaites L, Rey JM, Hazell PL, Graetz BW, Baghurst P (2002). Health-related quality of life of children and adolescents with mental disorders. J Am Acad Child Adolesc Psychiatry.

[2] Patalay P, Sharpe H, Wolpert M (2015). Internalising symptoms and body dissatisfaction: untangling temporal precedence using cross-lagged models in two cohorts. J Child Psychol Psychiatry.

[3] Hofstra MB, Van der Ende JAN, Verhulst FC (2000). Continuity and change of psychopathology from childhood into adulthood: a 14-year follow-up study. J Am Acad Child Adolesc Psychiatry.

[4] Copeland WE, Shanahan L, Costello E, Angold A (2009). Childhood and adolescent psychiatric disorders as predictors of young adult disorders. Arch Gen Psychiatry.

[5] Kessler RC, Berglund P, Demler O, Jin R, Merikangas KR, Walters EE (2005). Lifetime prevalence and age-of-onset distributions of DSM-IV disorders in the national comorbidity survey replication. Arch Gen Psychiatry.

[6] Department of Health (2015). Future in mind: promoting, protecting and improving our children and young people’s mental health and wellbeing.

[7] Weare K, Nind M (2011). Mental health promotion and problem prevention in schools: what does the evidence say?. Health Promot Int.

[8] Gulliver A, Griffiths KM, Christensen H (2010). Perceived barriers and facilitators to mental health help-seeking in young people: a systematic review. BMC Psychiatry.

[9] Schiller Y, Schulte-Körne G, Eberle-Sejari R, Maier B, Allgaier AK (2014). Increasing knowledge about depression in adolescents: effects of an information booklet. Soc Psychiatry Psychiatr Epidemiol.

[10] Clement S, Schauman O, Graham T, Maggioni F, Evans-Lacko S, Bezborodovs N, Morgan C, Rüsch N, Brown J, Thornicroft G (2015). What is the impact of mental health-related stigma on help-seeking? A systematic review of quantitative and qualitative studies. Psychol Med.

[11] Kelly CM, Jorm AF, Wright A (2007). Improving mental health literacy as a strategy to facilitate early intervention for mental disorders. Med J Aust.

[12] Wolpert M, Humphrey N, Deighton J, Patalay P, Fugard AJ, Fonagy P, Belsky J, Panos V (2015). An evaluation of the implementation and impact of England’s mandated school-based mental health initiative in elementary schools. Sch Psychol Rev.

[13] Wolpert M, Deighton J, Patalay P, Martin A, Fitzgerald-Yau N, Demir E, Fugard A, Belsky J, Fielding A, Fonagy P (2011). Me and my school: findings from the national evaluation of Targeted Mental Health in Schools.

[14] Stallard P, Simpson N, Anderson S, Carter T, Osborn C, Bush S (2005). An evaluation of the FRIENDS programme: a cognitive behaviour therapy intervention to promote emotional resilience. Arch Dis Child.

[15] Seligman MEP, Ernst RM, Gillham J, Reivich K, Linkins M (2009). Positive education: positive psychology and classroom interventions. Oxf Rev Educ.

[16] Montgomery AA, Peters TJ, Little P (2003). Design, analysis and presentation of factorial randomised controlled trials. BMC Med Res Methodol.

[17] Department for Education (2009) Schools, pupils and their characteristics: January 2009. http://webarchive.nationalarchives.gov.uk/20120504203418/http://education.gov.uk/rsgateway/DB/SFR/s000843/index.shtml. Accessed 26 Jan 2016

[18] Deighton J, Tymms P, Vostanis P, Belsky J, Fonagy P, Brown A, Martin A, Patalay P, Wolpert M (2013). The development of a school-based measure of child mental health. J Psychoeduc Assess.

[19] Patalay P, Deighton J, Fonagy P, Vostanis P, Wolpert M (2014). Clinical validity of the Me and My School questionnaire: a self-report mental health measure for children and adolescents. Child Adolesc Psychiatry Ment Health.

[20] The KIDSCREEN Group Europe (2006). The KIDSCREEN questionnaires––quality of life questionnaires for children and adolescents. Handbook.

[21] StataCorp. (2011). Stata statistical software: release 12.

[22] West BT, Welch KB, Galecki AT (2007). Linear mixed models: a practical guide using statistical software.

[23] Jorm AF, Griffiths KM, Christensen H, Parslow RA, Rogers B (2004). Actions taken to cope with depression at different levels of severity: a community survey. Psychol Med.

[24] Jonsson U, Alaie I, Parling T, Arnberg FK (2014). Reporting of harms in randomized controlled trials of psychological interventions for mental and behavioral disorders: a review of current practice. Contemp Clin Trials.

