# Supplementary material for: Use, acceptability and impact of booklets designed to support mental health self-management and help seeking in schools: results of a large randomised controlled trial in England
Source: Eur Child Adolesc Psychiatry. 2016 Jul 21;26(3):315–24. doi: 10.1007/s00787-016-0889-3 (PMC5323475; doi:10.1007/s00787-016-0889-3)
Supplement: Supplementary file 1 — Supplementary material 1 (DOCX 73 kb) [file 787_2016_889_MOESM1_ESM.docx]

Table S1: Descriptive statistics of impact outcomes at post-intervention

|  | Primary school | | | | Secondary school | | | |
| --- | --- | --- | --- | --- | --- | --- | --- | --- |
|  | TaMHS + booklet  n = 2662 - 2762 | Booklet only  n = 1219 - 1286 | TaMHS only  n = 2458 - 2598 | No intervention  n = 1409 - 1493 | TaMHS + booklet  n = 2456 - 2504 | Booklet only  n = 932 - 948 | TaMHS only  n = 2298 - 2361 | No intervention  n = 718 - 738 |
| Emotional (m [sd]) | 6.90 (3.47) | 6.73 (3.38) | 6.87 (3.52) | 6.84 (3.52) | 5.67 (3.34) | 5.79 (3.43) | 5.37 (3.22) | 5.65 (3.48) |
| Behavioural (m [sd]) | 3.10 (2.50) | 3.18 (2.56) | 3.20 (2.62) | 3.09 (2.50) | 3.26 (2.43) | 3.29 (3.40) | 3.15 (2.40) | 3.32 (2.52) |
| Quality of life (m [sd]) | 31.17 (4.77) | 31.06 (4.92) | 31.04 (4.85) | 3.96 (4.79) | 29.9 (4.17) | 29.5 (4.33) | 29.9 (4.15) | 29.9 (4.30) |
| Counsellor % yes (n) | 40.1 (1052) | 40.3 (491) | 46.5 (1143) | 37.8 (532) | 24.3 (600) | 21.5 (203) | 20.3 (473) | 23.2 (170) |
| Peer mentor % yes (n) | 38.5 (1026) | 40.0 (495) | 37.6 (935) | 35.1 (503) | 19.1 (467) | 21.7 (202) | 19.0 (467) | 17.8 (128) |
| Other help % yes (n) | 56.3 (1516) | 59.2 (745) | 58.0 (1468) | 55.4 (804) | 29.5 (726) | 29.4 (275) | 27.0 (622) | 29.1 (211) |
